# Communication between gut microbiota-derived metabolites and the tumor microenvironment

**DOI:** 10.3389/fimmu.2025.1649438

**Published:** 2025-10-15

**Authors:** Xinyi Hu, Bo Li, Yuanqing Li, Yushan Liang, Tingting Huang

**Affiliations:** ^1^ Department of Radiation Oncology, First Affiliated Hospital of Guangxi Medical University, Nanning, China; ^2^ Department of Otorhinolaryngology and Head and Neck Surgery, First Affiliated Hospital of Guangxi Medical University, Nanning, China; ^3^ Guangxi Key Laboratory of Early Prevention and Treatment for Regional High Frequency Tumor, Guangxi Medical University, Nanning, China

**Keywords:** gut microbiota, tumor microenvironment, gut microbiota-derived metabolites, cancer immunotherapy, immune cells, crosstalk

## Abstract

The gut microbiota has been increasingly recognized as a critical player in maintaining human health and influencing disease development. The tumor microenvironment (TME) is pivotal in tumor development and progression, comprising immune cells, stromal elements, extracellular matrix components, and cytokines. Recent studies have highlighted the promising potential of gut microbiota-derived metabolites (e.g., short-chain fatty acids, bile acids, polyamines, and tryptophan derivatives) to reshape the TME in various ways, generating significant interest for the development of novel therapeutic strategies. Beyond their established effects on traditional cancer treatments, emerging evidence suggests that microbiome-based interventions can substantially enhance cancer immunotherapy. However, the variable role of gut microbiota in modulating therapeutic responses complicates the prediction of clinical outcomes. Therefore, understanding the crosstalk between the gut microbiota and the TME is crucial and holds promise for the development of personalized and comprehensive cancer management strategies. This review aims to summarize the reciprocal regulatory mechanisms between gut microbiota-derived metabolites and the TME, and to explore how these interactions can be leveraged to improve cancer immunotherapy.

## 1 Introduction

The gut microbiota is a complex ecosystem of microorganisms in the gastrointestinal tract, importantly contributing to the maintenance of health and, when disrupted, to the development of disease (1). The tumor microenvironment (TME), as a dynamic ecosystem, encompasses the intricate cellular and acellular surroundings in which tumor cells proliferate, invade, and metastasize, comprising various components (e.g., immune cells, stromal elements, extracellular matrix components, and cytokines) that closely interact with the tumor cells (2). The crosstalk between the gut microbiota and the TME is increasingly recognized as an important factor in modulating cancer development, progression, and treatment response (3).

Evidence has accumulated to suggest different metabolites produced by the gut microbiota, including short-chain fatty acids (SCFAs), bile acids, polyamines, and tryptophan derivatives (4), as important mediators facilitating the communication between the gut microbiota and the TME. Specifically, SCFAs, as key metabolites of gut microbiota, are produced via the fermentation of undigested dietary fiber by specific intestinal anaerobic microbial communities and exhibit the highest concentration within the gut, primarily consisting of acetate, propionate, and butyrate (5). Bile acids are primarily synthesized in the liver from cholesterol to modulate host physiology and immune functions, and further interact with the gut microbiota to undergo various biotransformation, generating secondary bile acids (e.g., deoxycholic acid [DCA], lithocholic acid [LCA]) (6, 7). Furthermore, polyamines, including spermine, spermidine and putrescine, derive mainly from dietary protein, which serves as the major source of intestinal polyamines (8), and tryptophan derivatives are described as several indole-derivatives produced by gut microflora through catabolism of dietary tryptophan in the colon (9).

It has been shown that the TME interacts with different microbial metabolites to modulate tumorigenesis, immune evasion, and therapeutic responses (3), whereas microbial metabolites are known to modulate critical pathways in the TME such as immune cell differentiation, cytokine secretion, and tumor cell behavior (10). Conversely, tumor-associated inflammation and metabolic reprogramming within the TME can also influence the composition of the gut microbiota and alter metabolite synthesis (11). Understanding this reciprocal interplay between the gut microbiota and the TME shows therefore significant potential for targeting microbial metabolites to reshape the TME and improve cancer outcomes (12).

Cancer immunotherapy has rapidly evolved, offering transformative treatment options for patients; however, significant challenges, such as immune resistance and immune-related adverse events (irAEs), continue to limit its clinical efficacy and broader application (13). The manifestations of irAEs range from mild side effects to life-threatening complications, depending on factors such as the affected organ, tumor histology, and individual patient characteristics (13). IrAEs often affect the gut, skin, liver, and lungs, compromising treatment adherence and patient quality of life (14). Utilizing microbiota-host interactions to develop innovative strategies, such as fecal microbiota transplantation (FMT), pro- and prebiotics, and dietary interventions, with the aim of enhancing the efficacy of immunotherapy while reducing its side effects, is gaining momentum in cancer research (15). FMT is an innovative approach to restoring gut microbial homeostasis by transferring fecal matter from a healthy donor to a recipient (16). To reach the full potential of such strategies, a deeper understanding of specific microbial metabolites is essential for refining strategies for microbial modulation and identifying reliable biomarkers to guide personalized therapeutic interventions.

This review aims to synthesize current knowledge on the crosstalk between the gut microbiota and the TME, with a focus on summarizing the roles of gut microbiota-derived metabolites and how their interactions with the host TME may enhance cancer immunotherapy.

## 2 Gut microbiota-derived metabolites and modulation of the TME

Different gut microbiota-derived metabolites may play different regulatory roles in modulating the TME (15). SCFAs have been shown to significantly influence the TME by modulating the interactions between immune cells and the production of cytokines (17, 18). Bile acids and their metabolites could on the other hand influence the TME through regulating diverse immune cells (19). Polyamines may exhibit similar functional properties (8). Moreover, indole metabolites derived from tryptophan metabolism have demonstrated multifaceted roles within the TME, showcasing potential utility in both chemotherapy and immunotherapy (20).

### 2.1 T cells

T cells are integral to the evolvement and modulation of the TME (21). T cells engage in dynamic and context-dependent interactions within the TME, where T cells are tightly regulated by TME-derived signals (e.g., cytokines, metabolic stress, checkpoint molecules), ultimately dictating the efficacy of anti-tumor immunity or facilitating tumor immune evasion (22, 23). SCFAs help to shape T cell differentiation into either effector or regulatory phenotypes (24). CD8+ T cells function as core effector cells that mediate immune responses, acting as the primary target for various immunotherapeutic strategies (25). Specifically, SCFAs have been shown to enhance the functions of CD8+ T cell through inhibiting histone deacetylase (HDAC) and upregulating effector molecules, contributing to anti-tumor immune responses, particularly in colorectal cancer and gastric cancer (26–29). In addition to CD8+ T cells, SCFAs also exhibit diverse effects on other subsets of T cells. CD4+T cells exhibit an adaptive response to the immune microenvironment, ensuring the initiation of the optimal immune strategy in response to different types of immune challenges (30). Stimulated by specific environmental conditions, they differentiate into various cell subsets, such as Th1, Th2, Th17, and Treg cells, each assuming distinct roles in the immune response (31). Butyrate is known to attenuate CD4+ T cell activation by simultaneously inhibiting HDAC and G protein-coupled receptor 43 (GPR43) signaling, effectively suppressing the proliferation of Th1, Th17, and Th22 cells (32, 33). SCFAs have also been shown to promote regulatory T cells (Tregs), contributing to the maintenance of intestinal homeostasis and alleviation of certain pathological processes, such as abdominal aortic aneurysm (34–36). Moreover, propionate has been shown to inhibit IL-17 production by the γδ T cells during the inflammatory and tumorigenic processes (37).

Bile acids are also natural modulators of Th17/Treg balance. Lithocholic acid derivatives, such as 3-oxoLCA and isoalloLCA, could exhibit reciprocal effects by inhibiting Th17/Treg differentiation and enhancing Treg generation (38). Further, deoxycholic acid (DCA) may negatively influence the function of CD8+ T cells through suppressing the Ca^2+^-nuclear factor of activated T cells (NFAT)2 signaling, thereby facilitating immune evasion in colorectal cancer (39).

In addition to SCFAs and bile acids, other microbial metabolites can also modulate T cell responses. Polyamine metabolism is essential in T cell differentiation, e.g., spermidine has been shown to promote Treg differentiation and attenuating Th17 responses (40). Similarly, ornithine decarboxylase-dependent polyamine production is crucial for maintaining the fidelity of CD4+ T cells (41). Finally, tryptophan derivatives have been suggested to affect the number of Treg cells and induce apoptosis in Th1/Th17 cells (42, 43).

In summary, metabolites derived from the gut microbiota can intricately regulate T cell responses through epigenetic, metabolic, and receptor-mediated mechanisms, presenting significant opportunities for therapeutic interventions in cancer. Future studies should explore tissue-specific effects, dose-dependent outcomes, and translational applicability of these metabolite-based therapies.

### 2.2 B cells

B cells exert multifaceted roles that collectively shape anti-tumor immunity and correlate with prognostic outcomes, encompassing antigen presentation, antibody production, organization of tertiary lymphoid structures, and regulation via immunosuppressive B regulatory cells (Bregs) (44). Gut microbiota-derived metabolites can influence B cell responses through multiple metabolic and signaling pathways. The enhancing effect of SCFAs on B cell antibody production essentially works by reshaping the energy metabolism pathway of B cells and indirectly consolidating the intestinal immune barrier (45). SCFAs enhance antibody production in B cells by increasing levels of intracellular acetyl-CoA and subsequently stimulating oxidative phosphorylation, glycolysis, and fatty acid synthesis, thus bolstering intestinal and systemic immunity (46). SCFAs can also function as epigenetic regulators of B cell differentiation and activity, influencing both the homeostatic and pathogen-specific antibody responses (47). For instance, butyrate has been shown to promote the differentiation of IL-10-producing (IL-10+) Bregs, a process associated with the inhibition of HDAC3 activity and the reduction of mitochondrial oxidative stress (48). Furthermore, butyrate can enhance the immunosuppressive capabilities of Bregs, important for maintaining immune tolerance (49).

Nonetheless, it is important to note that the immunomodulatory effects of SCFAs on B cells are dose dependent. Low levels of butyrate and propionate have been shown to moderately enhance class-switch DNA recombination in B cells whereas higher levels can inhibit activation-induced cytidine deaminase and Blimp1 expression, ultimately suppressing class-switch DNA recombination (CSR) and plasma cell differentiation (50). Careful clarification of such nuanced, dose-dependent effects is therefore important for effectively harnessing SCFAs in the therapeutic modulation of B cell responses.

### 2.3 Macrophages

Macrophages are pivotal components of the TME, and their polarization states are intricately regulated by metabolites derived from the gut microbiota. M1 macrophages exhibit tumoricidal activity and reinvigorates cytotoxic T-cell responses, whereas M2 macrophages foster immune evasion and tumor progression (51, 52). Different metabolites exert distinct and multifaceted effects on macrophages. For instance, SCFAs have been shown to modulate the dynamic balance of M1/M2 macrophages by suppressing M1 macrophage polarization and promoting M2 macrophage polarization, thereby participating in tumor-related pathological processes (53–55). Interestingly, *B.thetaiotaomicron*-derived acetic acid was proved to improve the polarization of M1 macrophages and further promotes the function of cytotoxic CD8+ T cells, ultimately inhibiting the growth of hepatocellular carcinoma tumors (56).

Other microbial metabolites also influence macrophage function. Trimethylamine N-oxide (TMAO), a metabolite produced by the gut microbiota, was shown to promote M1 macrophage polarization via NOD-like receptor protein 3 (NLRP3) inflammasome activation (57) and enhance the cytotoxic capacity of M1 macrophages against tumor cells (58). Moreover, recent studies have suggested that indole-3-acetic acid, a tryptophan-derived metabolite, promotes the IL-35 production in macrophages and other immune cells, subsequently alleviating intestinal inflammation and suppressing tumorigenesis (59).

These findings collectively highlight the complex and context-specific nature of microbial metabolite-mediated regulation of macrophages. Further research is needed to elucidate tissue-specific mechanisms, enabling more effective therapeutic modulations of macrophages in cancer.

### 2.4 Other immune cells

In addition to T cells, B cells, and macrophages, gut microbiota-derived metabolites also modulate the functions of other immune cell types that are important to the immunological landscape of the TME.

For instance, dendritic cells (DCs) uniquely orchestrate antitumor responses through their specialized capacity for cross-presenting tumor antigens to naïve T cells (60). SCFAs regulate the expression of genes related to inflammation and immune-cell recruitment through HDAC inhibition, resulting in particularly strong modulatory effects in DCs and enhanced anti-inflammatory activity (61). Moreover, SCFAs promote dendrite elongation in DCs, assisting antigen uptake and key processes for effective T cell activation (62).Secondary bile acids have also been shown to inhibit DC activity through inhibiting nuclear factor κB (NF-κB)-mediated activation via the TGR5-cAMP-PKA axis (63).

Myeloid-derived suppressor cells (MDSCs) represent a heterogeneous population of pathologically responded neutrophils and monocytes, which exhibit a strong correlation with unfavorable clinical prognoses in cancer and immune responses (64–67). Butyrate has been shown to induce epigenetic and metabolic reprogramming in MDSCs, promoting their expansion and immunosuppressive capacity (68). In contrast, bile acid has been shown to recruit MDSCs and help mitigate excessive immunosuppression, via the cancer-associated fibroblast-CCL3/CCR1 axis (69).

Natural killer (NK) cells, as key innate effectors in anti-tumor immunity (70), are also modulated by SCFAs. Recent studies have shown that SCFAs can enhance the proliferation and function of NK cells by promoting the release of NK-derived extracellular vesicles and reducing the levels of anti-inflammatory cytokine IL-10, suggesting that SCFAs can contribute the anti-tumor NK cell responses (71). Finally, high levels of SCFAs have been shown to impair the migration and antiviral defense of neutrophils against human immunodeficiency virus, with potentially age- and sex-dependent regulatory characteristics (72). Moreover, butyrate and propionate can induce apoptosis and degranulation in basophils to modify basophil-mediated immune responses (73).

Collectively, these findings emphasize the important role of metabolites derived from the gut microbiota in regulating a wide array of immune cell types within the TME. Further research is warranted to delineate the specific molecular mechanisms by which these metabolites exert such function under different pathological conditions. Ultimately, these insights could guide the development of microbiota-targeted therapies aimed at reshaping the immune landscape in cancer (**Table 1**).

**Table 1:** Crosstalk of major gut microbiota-derived metabolites and the tumor immune microenvironment: clinical translational potential.

Gut Microbiota-Derived Metabolites	Targeting Immune Cells	Immune Effects	Associated Cancers	Potential for Clinical Translation	References
SCFAs	CD8^+^ T cells	1. Enhance the cytotoxic activity of CD8^+^ T against gastric cancer cells via the GPR109A/HOPX axis; 2. Up-regulate gastric cancer cell expression of SCFA receptors (GPR109A, GPR43) and antigen-processing genes (e.g., NLRC5, Tap1, Tap2)	Gastric cancer (GC)	1. Butyrate supplementation inhibits gastric tumorigenesis and progression in animal models; 2. *In vitro* studies demonstrate that butyrate suppresses gastric cancer cell proliferation and promotes apoptosis; 3. Fecal and serum SCFA (especially butyrate) levels are significantly low in GC patients.	(26)
SCFAs	CD8^+^ T cells	1. Activate CD8^+^ T cells to produce IFN-γ and granzyme and further up-regulates tumor MHC I expression to reinforce immune responses; 2. Activate the cGAS/STING pathway by inhibiting histone deacetylases (HDACs) to induce DNA damage in colorectal cancer cells, up-regulating chemokines (CCL5, CXCL10) and ISGs	Colorectal cancer (CRC)	1. *In vitro* studies demonstrate that SCFAs enhance tumor immunogenicity; 2. *In vivo* studies link higher SCFA levels with abundance of SCFA-producing gut microbiota.	(27)
SCFAs	CD8^+^ T cells	1. Promote CD8^+^ T cell production of IFN-γ and granzyme B in an ID2-dependent manner, enhancing cytotoxicity and antitumor activity; 2. Up-regulate IL-12 receptor expression, boosting CD8^+^ T cell responsiveness to IL-12 and promoting effector function; 3. Enhance oxaliplatin chemotherapy efficacy	CRC, lymphoma, colitis-associated CRC	1. Oral or intraperitoneal butyrate augments oxaliplatin efficacy in animal models; 2. Clinical data show higher serum butyrate in oxaliplatin-responsive cancer patients; 3. Preclinical combination of butyrate with anti-programmed cell death ligand 1 (PD-L1) immunotherapy enhances antitumor effects.	(28)
SCFAs	CD8^+^ T cells	Promote CD8^+^ T cell memory formation, modulates cellular metabolism, and sustains memory cell survival.	Indirectly participate in the tumor process	1. *In vitro* studies demonstrate that CD8^+^ T cells treated with butyrate show stronger expansion and IFN-γ production; 2. High-fiber diet increases circulating SCFAs and enhances recall responses of memory CD8^+^ T cells.	(29)
SCFAs	CD4^+^ T cells	Inhibit CD4^+^ T cell activation and pro-inflammatory cytokine (IFN-γ, IL-17) production in a dose-dependent manner via HDAC inhibition and GPR43 activation, affecting Th1, Th17, and Th22	Indirectly participate in the tumor process	1. High-fiber diet increases butyrate and alleviates gut inflammation in animal models; 2. Fecal/tissue butyrate levels reflect intestinal immune homeostasis; 3. Butyrate enemas or HDAC inhibitors are under preclinical/early clinical investigation as adjuvant therapy for immune-checkpoint blockade (ICB) in IBD.	(32, 33)
SCFAs	Tregs	1. Promote peripheral Treg generation; 2. Stabilize FOXP3 expression via HDAC inhibition and increase histone acetylation at the FOXP3 locus, enhancing Treg function	Indirectly participate in the tumor process	Dietary SCFA or prebiotic supplementation proposed as a theoretical basis for Treg modulation in autoimmune diseases.	(34)
Propionate	Tregs	Specifically expand colonic lamina propria Tregs, down-regulate CD69 expression, and promote Treg trafficking via draining lymph nodes and blood to sites of atherosclerosis.	Indirectly participate in the tumor process	SCFA supplementation (e.g., propionate) or modified starches to increase intestinal SCFA levels proposed as a preventive strategy for Abdominal Aortic Aneurysm at-risk populations.	(35)
SCFAs	Th17/Tregs	1. Gut dysbiosis reduces propionate, skewing Th17/Treg balance (Th17↑, Treg↓); 2. Propionate supplementation restores Th17/Treg equilibrium by promoting Treg and suppressing Th17 differentiation via GPR43 activation and HDAC6 inhibition.	Indirectly participate in the tumor process	Propionate supplementation or microbiota modulation proposed as a novel immunomodulatory approach for chronic prostatitis/chronic pelvic pain syndrome (CP/CPPS).	(36)
SCFAs	Γδ T cells	Directly inhibit γδ T cell IL-17 and IL-22 production via HDAC inhibition	CRC	SCFAs (especially propionate) proposed as potential targets for modulating γδ T cell function in IBD and CRC.	(37)
SCFAs	B cells	Drive B cell differentiation into IL-10^+^ IgM^+^ regulatory plasma cells	Indirectly participate in the tumor process	Butyrate analogues under investigation as adjunct therapy for metabolic syndrome.	(45)
SCFAs	B cells	1. Butyrate/propionate promotes the differentiation of IL-10^+^ IgM^+^ regulatory plasma cells and reduces pathogenic class switching via HDAC inhibition. 2. Acetate promotes the generation of Bregs and inhibits pro-inflammatory cytokines (e.g., TNFα); Butyrate reduces mitochondrial reactive oxygen species (ROS) in B cells via HDAC3 inhibition to maintain Breg homeostasis. 3. Butyrate induces the production of TGF-β and retinoic acid (RA), promoting IgA class switching in B cells and enhancing the intestinal barrier function.	CRC	1. *In vitro* studies clarify the regulatory effects of SCFAs on B cell differentiation and antibody production. 2. HDAC inhibitors (e.g., butyrate analogs) reduce autoreactive plasma cells in animal models.	(46)
SCFAs	B cells	1. Enhance B cell metabolism and provide energy and material basis for plasma cell differentiation; 2. Promote the production of IgA and IgG, enhancing the immune response against pathogens; 3. Indirectly regulate T cells by increasing the number of Tfh cells, promoting germinal center reactions, and assisting B cell antibody production.	Indirectly participate in the tumor process	1. Animal studies have confirmed that a high-fiber diet/SCFA supplementation can enhance antibody levels and SCFAs regulate B cell functions through metabolic regulation and HDAC inhibition; 2. Antibiotic treatment can eliminate the antibody-promoting effect of SCFAs, confirming microbiota dependence.	(47)
Butyrate	Bregs	Promote IL-10 expression in Bregs, enhance their suppressive function, and inhibit germinal-center B cells and plasmablast differentiation	Indirectly participate in the tumor process	Butyrate supplementation alleviates intestinal inflammation.	(48)
SCFAs	Bregs	Enhance the suppressive function of Bregs, increase IL-10 secretion, and reduce the differentiation of plasmablasts, decrease the production of pro-inflammatory cytokines (TNFα, IL-6, MCP-1)	No associated cancers mentioned	1. Verified the anti - inflammatory effect of butyrate supplements in animal models; 2. Fecal butyrate levels are decreased in rheumatoid arthritis patients and are positively correlated with peripheral blood Bregs.	(49)
SCFAs	B cells	Dose-dependently modulates B cells: low concentrations (50–200 µM) mildly increase AID expression and class-switch recombination (CSR); high concentrations (≥400 µM) inhibit AID, Blimp1, CSR, somatic hypermutation, and plasma cell differentiation	Indirectly participate in the tumor process	Modulating butyrate levels inhibits autoantibody production and alleviates lupus symptoms in animal models.	(50)
SCFAs	M2 Macrophages	1. Trigger TLR3-induced autophagy in cancer cells, activating NF-κB and MAPK pathways and enhancing migration and invasion, autophagy induces CCL20 release; 2. CCL20 can recruit macrophages into the tumor microenvironment (TME) and polarizes them toward pro-tumor M2 Macrophages, further enhancing prostate cancer invasiveness.	Prostate cancer	1. Preclinical studies establish SCFAs from Castration-Resistant Prostate Cancer-associated microbiota as key mediators linking dysbiosis to tumor progression; 2. CCL20 identified as a potential prognostic biomarker for prostate cancer.	(53)
SCFAs	Macrophages	1. Inhibit LPS-induced M1 polarization (↓iNOS, TNF-α) and promote IL-4–induced M2 polarization (↑Arg-1, IL-10); 2. Down-regulate TLR4, MyD88, NF-κB, and suppress alcohol-induced liver injury	Indirectly participate in the tumor process	1. Preclinical evidence shows inulin increases intestinal SCFAs; 2. SCFAs exert anti-Alcoholic liver disease (ALD) effects by modulating M1/M2 macrophage balance, providing rationale for inulin/SCFA-based ALD prevention and therapy.	(54)
SCFAs	Macrophages	1. Modulate M1/M2 balance, ↓M1, ↑M2; reduce serum pro-inflammatory cytokines (IL-12p70, TNF-α, CXCL1); 2. ↑tight-junction proteins (ZO-1, occludin), restore barrier function	Indirectly participate in the tumor process	Positive correlations between SCFAs and bone-metabolism indices suggest novel gut-targeted osteoporosis therapy.	(55)
Acetate	Macrophages	1. Promote pro-inflammatory M1 polarization (↑CD86, iNOS; ↓CD163, ARG1) via histone acetylation-driven ACC1 transcription and increase fatty-acid synthesis; 2. M1 macrophages enhance CD8^+^ T cell function (↑IFN-γ, granzyme B), increasing cytotoxicity against hepatocellular carcinoma (HCC) cells.	HCC	1. Preclinical studies show *B.thetaiotaomicron*-derived acetate inhibits HCC growth via immune-microenvironment modulation; 2. Acetylation inhibitors (e.g., curcumin) block acetate-mediated tumor suppression, offering epigenetic-targeted HCC therapy.	(56)
SCFAs	Dendritic cells (DCs)	↓ pro-inflammatory cytokine secretion (IL-6, IL-12) by DCs and ↓ chemokines (CXCL9, CXCL10, CXCL11)	Potentially applicable to inflammation-associated cancers	Butyrate proposed as an anti-inflammatory agent for modulating DC function.	(61)
SCFAs	DCs	1. Induce DC dendrite elongation via HDAC inhibition, promoting actin polymerization; 2. Enhance antigen uptake and presentation	Indirectly participate in the tumor process	Clinical application not yet addressed.	(62)
Butyrate	Myeloid-derived suppressor cells (MDSCs)	1. Promote MDSC suppressive function via fatty-acid β-oxidation (FAO) metabolic reprogramming; 2. Enhances T cell inhibition by MDSCs	Indirectly participate in the tumor process	1. Butyrate alleviates cholangitis in animal models; 2. Positive correlation observed between butyrate levels and MDSC function/treatment response in humans.	(68)
SCFAs	Natural killer (NK) cells	Promote the release of extracellular vehicles (EVs), significantly reduce the secretion of the anti - inflammatory cytokine IL-10, and indirectly weaken the pro - tumor effect of IL-10.	Multiple myeloma	1. Enhancing the cytotoxicity of NK cells through SCFA preconditioning can optimize the effect of NK cell immunotherapy; 2. In combination with ICB, chemotherapy, etc., SCFAs may improve treatment response and reduce drug resistance.	(71)
SCFAs	Neutrophils	1. Butyrate ↓CD66b, ↑CD16 and CD62L, yielding a low-activation, long-lived mature phenotype; propionate ↑CD54 and CXCR4, inducing a senescent phenotype; 2. Acetate and butyrate suppress neutrophil migration *in vitro*; propionate alters migratory phenotype (↑CD62L, CD54) without affecting migration.	Indirectly participate in the tumor process	*In vitro* studies demonstrate that pathological concentrations of SCFAs impair the anti-HIV function of neutrophils.	(72)
Butyrate, propionate	Basophils	1. Induce CD69 expression and shift cytokine secretion (↓IL-4, ↑IL-13) via HDAC inhibition; 2. Induce basophil apoptosis even in the presence of IL-3 (apoptosis inhibition); 4. Enhance IgE-mediated degranulation	Indirectly participate in the tumor process	Mechanisms of HDAC-mediated basophil modulation by propionate and butyrate are clarified.	(73)
3-oxoLCA, isoalloLCA	Th17/Tregs	1. 3-oxoLCA directly bind the Th17 transcription factor RORγt, inhibiting its activity and reducing IL-17 secretion; 2. IsoalloLCA promotes mitochondrial ROS generation to induce Treg differentiation.	Indirectly participate in the tumor process	1. Oral 3-oxoLCA reduces intestinal Th17 cells in animal models; 2. Combined 3-oxoLCA and isoalloLCA feeding increases Tregs and alleviates colitis in animal models.	(38)
Bile acids	CD8^+^ T cells	Inhibit CD8^+^ T cell function by enhancing PMCA activity, suppressing Ca²^+^-NFAT2 signaling, and reducing IFN-γ, TNF-α, and granzyme B secretion.	CRC	1. Bile acid sequestrants (e.g., cholestyramine) lower DCA and inhibit tumor growth; 2. Fecal DCA concentration and microbial baiF gene (key for DCA synthesis) abundance are potential CRC risk biomarkers; 3. Polyamine blockade therapy combined with PD-1 inhibitors may reverse “cold tumor” microenvironment.	(39)
Secondary bile acids	DCs	Inhibit NF-κB activation via the TGR5–cAMP–PKA pathway, reducing secretion of pro-inflammatory factors (IL-1β, IL-6, TNF-α).	Indirectly participate in the tumor process	Oral DCA/LCA alleviates experimental autoimmune uveitis (EAU) in animal models.	(63)
Bile acids	MDSCs	1. Promote MDSC infiltration into liver metastases and suppress T cell activation; 2. MDSC-derived CCL2 attenuates immunosuppression via CCR2 signaling.	Colorectal cancer liver metastasis (CRLM)	Potential targets (TGR5, CCL3, CCR1) proposed but remain preclinical.	(69)
Spermidine	CD4^+^ T cells	Inhibit CD4^+^ T cell via MAPK/ERK pathway, reduce activation marker CD69 and IL-2 production, decrease Th1 and Th17 differentiation	Indirectly participate in the tumor process	*In vitro* studies demonstrate that Spermidine show preventive and therapeutic effects, offering a potential strategy for multiple sclerosis requiring further preclinical and clinical validation.	(40)
L-Tryptophan (L-Trp)	Tregs	Promote Treg homing to the colon via the AhR-GPR15 pathway and increase colonic Tregs	Potentially reduce colitis-associated cancer risk	L-Trp supplementation is proposed as a non-invasive preventive therapy for ulcerative colitis (UC).	(42)
Indole-3-propionic acid (IPA)	Th1/Th17	Bind HSP70, trigger mitochondrial-dependent apoptosis in Th1/Th17 cells	Potentially reduce IBD-associated CRC risk	Oral IPA alleviates colitis in animal models and is proposed as a therapeutic strategy for IBD.	(43)
Indole-3-acetic acid (IAA)	Macrophages	Induce IL-35 expression, promote Treg, Breg, and M2 macrophage differentiation, and inhibit Th1 differentiation.	Colitis-associated CRC	IAA levels are low in CRC patients, suggesting diagnostic or preventive value.	(59)
TMAO	M1 Macrophages	Activate NLRP3 inflammasome, promote mitochondrial ROS, activate NF-κB, induce M1 macrophage polarization, and enhance Th1 and Th17 differentiation	Relevant to Graft-versus-host disease (GVHD) after hematopoietic stem-cell transplantation for hematologic malignancies	Choline analogue can alleviate GVHD, suggesting therapeutic potential for dietary interventions or drugs targeting the TMAO pathway.	(57)
TMAO	Macrophages	Activate IFN-I pathway, promote M1 macrophage polarization, and enhance CD8^+^ T cell function	Pancreatic ductal adenocarcinoma, melanoma	1. Higher TMAO levels correlate with improved long-term survival and immunotherapy response; 2. Dietary choline supplementation or adoptive transfer of TMAO-conditioned macrophages shows therapeutic potential.	(58)

## 3 Gut microbiota-derived metabolites and cancer immunotherapy

The dynamic interplay between the gut microbiota and the immune system forms the foundation for how gut microbiota-derived metabolites influence immune functions and disease outcomes. Leveraging this interaction offers a promising strategy to enhance immune responses and alleviate immunological disorders. This section examines the translational implications of host-microbiota crosstalk in improving the efficacy of cancer immunotherapy.

### 3.1 Immune checkpoint blockade therapy

ICB therapy has revolutionized cancer immunotherapy by targeting inhibitory pathways, such as programmed cell death protein 1 (PD-1)/programmed cell death ligand 1 (PD-L1) and cytotoxic T-lymphocyte-associated protein 4 (CTLA-4), which regulate immune system homeostasis under physiological conditions while tumors exploit to escape immune surveillance (74, 75). Through blocking these checkpoints, ICB reactivates T cell-mediated anti-tumor responses (76). Emerging evidence has indicated that the gut microbiota significantly influences the efficacy of ICB therapy, whereas the microbial diversity and composition of the gut microbiota contribute importantly to treatment outcomes (77). For instance, melanoma patients responding to anti-PD-1 therapy have been shown to exhibit higher microbial diversity and an enrichment of specific bacterial taxa, compared with non-responders, in the gut microbiota (78).

Microbial metabolites can also modulate ICB therapy. Phenylacetylglutamine (PAGln) has been shown to negatively correlate with ICB efficacy (79), whereas TMAO was shown to synergize with immune checkpoint inhibitors to reduce tumor burden and improve survival in a pancreatic ductal adenocarcinoma model (58). The role of microbial metabolites in immunotherapy is not necessarily monolithic. For example, tryptophan metabolites have been shown to exert dual roles, namely that they enhance ICB efficacy through modulating tumor-associated macrophages but also promote tumor progression via IL4I1-mediated AhR activation (80, 81). A similarly complex picture has been noted for SCFAs. For instance, high levels of butyrate have been suggested to impair anti-CTLA-4 therapy by increasing the frequencies of Tregs and reducing tumor-specific T cell infiltration (82).

Regardless, existing studies suggest a potentially central role of the gut microbiota and its derived metabolites in modulating the efficacy of ICB therapy. Improved understanding of the interactions between different microbial metabolites and the TME helps to develop personalized strategies to enhance therapeutic responses (83).

### 3.2 Gut microbiota-derived metabolites and adverse events of immunotherapy

Enhancing the efficacy of ICB therapy is utmost important; however, mitigating irAEs is equally critical. The gut microbiota and its derived metabolites have been implicated to modulate the severity of irAEs, particularly in the gastrointestinal tract (84, 85). The gut-liver axis further exemplifies how microbiota-mediated immune regulation can influence systemic toxicity profiles (86).

Specific microbial metabolites have been linked to the susceptibility of irAEs. For instance, menaquinone has been suggested as a potential modulator of adverse immune responses (87) whereas butyrate has been shown to reinforce intestinal barrier integrity and ameliorate immune checkpoint inhibitors (ICIs)-induced colitis (88). Indole-3-carboxaldehyde, a tryptophan metabolite, may exert similar regulatory effects as butyrate (89). To better identify strategies to prevent or alleviate irAEs, in-depth characterization of key microbiota-immune crosstalk pathways is needed.

### 3.3 Fecal microbiota transplantation

FMT has been shown to reprogram the gut microbiota and the TME among immunotherapy-refractory patients (68, 90) and to restore anti-PD-1 sensitivity among patients with refractory melanoma and other malignancies (91–94). Moreover, FMT has been shown to increase the production of SCFAs and facilitate the infiltration and activation of immune cells to the TME, thereby improving therapeutic efficacy (95). The potential of FMT has also been suggested in hepatocellular carcinoma, particularly in managing intrahepatic metastases (96). Although the potential of FMT as an add-on therapeutic strategy for immunotherapy of diverse cancer types is clear, challenges exist regarding donor screening, protocol standardization, and potential side effects (16, 97). Rigorously designed clinical trials and preclinical models are needed to illuminate the trade-off between benefits and potential harms (90).

### 3.4 Probiotics and prebiotics

Probiotics and prebiotics represent targeted strategies to modulate the composition and function of the gut microbiota (98). As a vital supplementary treatment method, probiotics have been proved to restore the microbial imbalance caused by cancer treatment, thereby alleviating gastrointestinal adverse reactions and stimulating the immune system to fight against tumor cells (99, 100). *Clostridium butyricum*, for instance, can suppress colorectal cancer associated with colitis and enhance efficacy of ICB therapy (101–104). Prebiotics, which are selectively utilized by host microorganisms (e.g., glucans and fructans), support the colonization and functions of probiotics and enhance the production of SCFAs (105). For example, pectin has been shown to selectively enrich SCFA-producing taxa (e.g., Bifidobacterium and Lactobacillus), contributing to an immunostimulatory TME (106). Together, prebiotics and probiotics modulate the gut microbiota to promote host health, with overlapping mechanisms such as immune regulation and gut barrier improvement (107). However, as various factors (e.g., strain specificity, host health status, and diet) could influence outcomes of pro- or prebiotics use, individualized approaches and therapeutic guidelines are urgently needed (98, 108). Precision probiotics, tailored to specific microbiome phenotypes, may optimize therapeutic efficacy by promoting the growth of beneficial metabolite-producing microbes (109). Clinical validation and standardized guidelines are therefore essential for the integration of such interventions to personalized oncology (110).

### 3.5 Dietary interventions

Dietary interventions targeting the gut microbiota have emerged as a non-invasive strategy to improve the immune status and support cancer immunotherapy (111). For example, high dietary cholesterol has been revealed to result in non-alcoholic fatty liver disease-related hepatocellular carcinoma (NAFLD-HCC) through dysbiosis of gut microbiota and metabolites and anticholesterol treatment has significant potential in preventing cancer (112). Furthermore, a high-fiber diet lays a solid immune foundation for strengthening the intestinal immune barrier and enhancing T cell activation to improve responses to anti-PD-1 therapy, promoting the proliferation of gut bacteria that produce SCFAs and increases endogenous SCFA levels (113, 114), especially propionates have been proved to alleviate lipid dysmetabolism and enhance immune homeostasis (115–117). Other microbial metabolites derived from dietary components also exhibit immunomodulatory properties. Polyamines (e.g., spermidine) derived from whole grains and fermented foods help to modulate T cell differentiation and contribute to gut immunity (118). Moreover, appropriate reduction in daily protein intake can enhance the enrichment of beneficial gut bacteria and modulate host health status through microbial-derived metabolites (119).

However, inter-individual microbiome variability and varying adherence to dietary interventions might influence efficacy (120). Successful clinical use of personalized dietary interventions will require a deeper phenotyping of individual microbiota profiles and a validation through rigorously designed clinical trials. Notably, given their relatively minimal side effects, the significant potential of dietary interventions in tumor immunotherapy represents a promising avenue for further exploration.

### 3.6 Emerging biomarkers for cancer immunotherapy

As the targeted modulation of the gut microbiota has emerged as an innovative therapy for cancer, the information encoded within the compositional and metabolic profiles of the gut microbiota is increasingly being harnessed to develop novel biomarkers for the prediction of risk and prognosis of cancer, indicating another important clinical utility of the gut microbiota (121). Intestinal microbiota exhibits a dynamic and real-time correlation with tumor progression and therapeutic interventions, enabling a more comprehensive and timely assessment of treatment efficacy compared to traditional biomarkers (122). Specifically, there appears to be notable heterogeneity between tumor types. Decreased abundance in specific probiotic species has been linked to a dysbiotic state associated with poor outcomes of colorectal cancer (123).

Gut microbiota metabolites also show potential for non-invasive screening and treatment response prediction (124). Reduced levels and decreased abundance of SCFA-producing bacteria have been shown to be correlated with risk markers in non-small cell lung cancer (84, 125), while secondary bile acids (e.g., deoxycholic acid) with elevated levels and increased abundance of related metabolizing bacteria can act as diagnostic markers in CRC patients (126). Moreover, tryptophan metabolites, particularly indoxyl sulfate (IS), appear to serve as key predictors for differentiating ruptured from unruptured intracranial aneurysms (127).

Nevertheless, owing to the individual variations caused by factors such as diet and antibiotic use, as well as the lack of standardized detection technologies, further verifying the reliability of microbial biomarkers are crucial for fully realizing clinical transformation (123).

## 4 Conclusions and future perspectives

**Figure 1:**
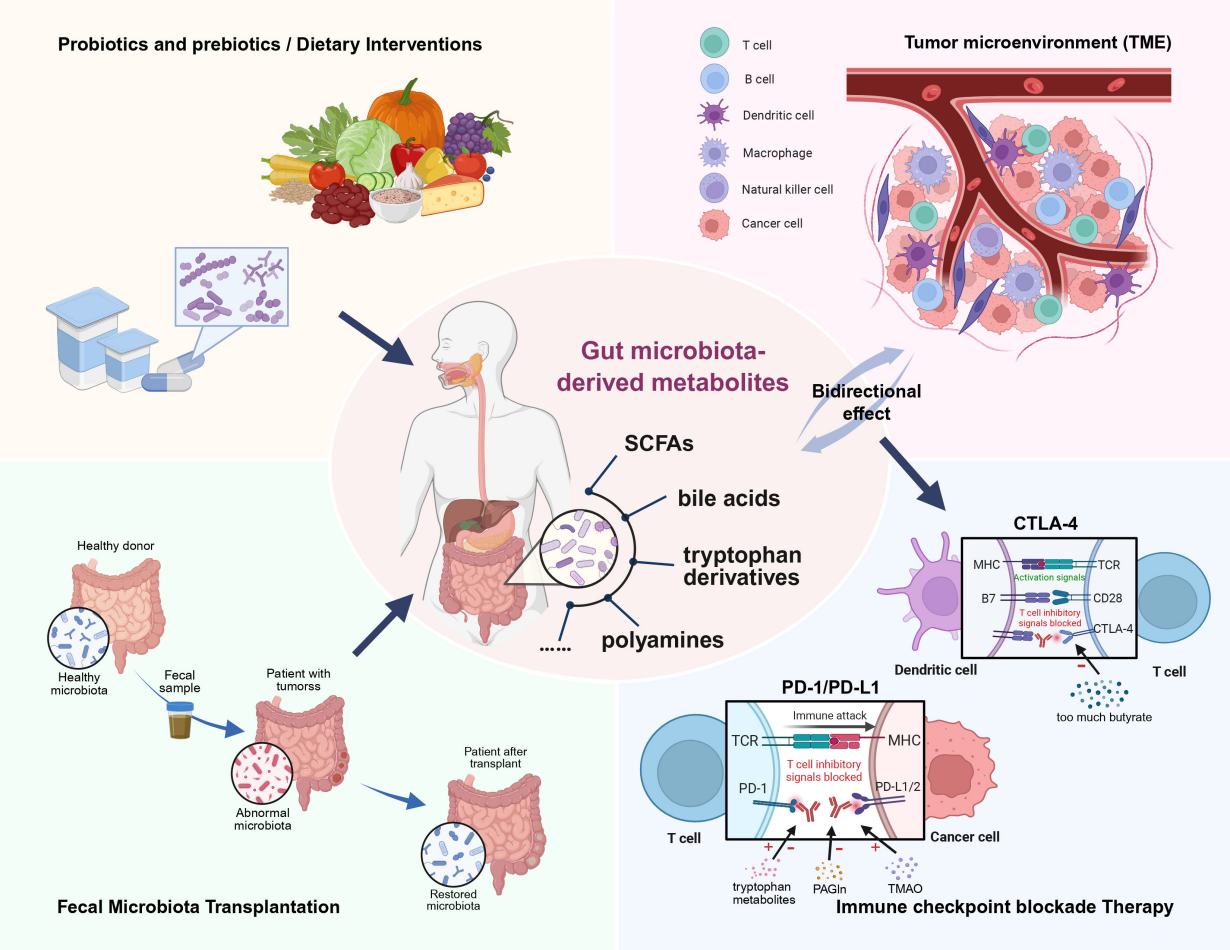
Gut microbiota-derived metabolites play a crucial role in modulating the tumor microenvironment (TME) and influencing the efficacy of cancer therapies. This review highlights the ability of various metabolites to mediate immune responses within the TME. Deciphering and harnessing this crosstalk holds significant promise for advancing cancer immunotherapy, particularly in supporting tailored immune checkpoint blockade (ICB) therapies that target specific molecules. Additionally, targeted fecal microbiota transplantation (FMT), along with other supportive measures such as probiotics, prebiotics, and dietary interventions, may help restore gut microbial homeostasis and its associated metabolic profiles, ultimately enhancing cancer therapy outcomes.

The gut microbiota plays a pivotal role in modulating the immune responses within the TME and shaping the efficacy of cancer therapies, especially immunotherapy. Investigating the therapeutic potential of gut microbiota-derived metabolites is an emerging frontier in precision oncology, presenting new opportunities to improve clinical outcomes of cancer patients. The convergence of microbiology, immunology, and oncology will facilitate a holistic paradigm shift in cancer care (128) (**Figure 1**).

Continued research is clearly needed to best translate bench-side discoveries into clinical applications (129). Innovative technologies and personalized strategies, such as AI-based identification of immunomodulatory gene targets (130), microbiota‐targeted nanomedicine via genetic engineering (131), and development of novel postbiotics or metabolite supplementation (132), could all potentially help improve the efficacy of cancer immunotherapy. Moreover, advanced metabolomics approaches – such as untargeted metabolomics or stable-isotope tracing – should also be leveraged to uncover additional microbiota-derived metabolites of relevance to the TME and efficacy of immunotherapy (133). In addition to the identification of novel metabolites, integrative use of advanced metagenomics and metatranscriptomics techniques can also help identify microbial genes and pathways critical for immune modulation (134, 135). Translational studies should on the other hand expand to include robust, well-powered clinical trials that evaluate different microbiota-targeted therapies such as engineered probiotics, synthetic microbial consortia, and postbiotic supplementation across diverse patient populations (136, 137). Finally, integrating microbiome interventions with emerging cancer therapies – such as CAR-T cells and cancer vaccines – also represents a promising new frontier (138).

Despite significant advancement, several challenges remain. The mechanisms by which microbial metabolites influence immune responses within the TME need further exploration, and their long-term health effects must be thoroughly evaluated. The complexity of host-microbiota interactions necessitates a comprehensive, systems-level research approach. Moreover, population-specific variability underscores the need for large-scale, diverse clinical studies. Personalized therapeutic strategies tailored to individual microbiota profiles could lead to substantial improvements in cancer care. Expanding clinical trial cohorts and ensuring adequate statistical power are essential for generalizing findings and implementing microbiota-based interventions across diverse populations.
