# Supplementary material for: Communication between gut microbiota-derived metabolites and the tumor microenvironment
Source: Front Immunol. 2025 Oct 15;16:1649438. doi: 10.3389/fimmu.2025.1649438 (PMC12568434; doi:10.3389/fimmu.2025.1649438)
Supplement: Supplementary file 1 [file Table1.docx]

Table 1 Crosstalk of Major Gut Microbiota-Derived Metabolites and the Tumor Immune Microenvironment: Clinical Translational Potential

| **Gut Microbiota-Derived Metabolites** | **Targeting Immune Cells** | **Immune Effects** | **Associated Cancers** | **Potential for Clinical Translation** | **References** |
| --- | --- | --- | --- | --- | --- |
| SCFAs | CD8⁺ T cells | 1. Enhance the cytotoxic activity of CD8⁺ T against gastric cancer cells via the GPR109A/HOPX axis; 2. Up-regulate gastric cancer cell expression of SCFA receptors (GPR109A, GPR43) and antigen-processing genes (e.g., NLRC5, Tap1, Tap2) | Gastric cancer (GC) | 1. Butyrate supplementation inhibits gastric tumorigenesis and progression in animal models; 2. In vitro studies demonstrate that butyrate suppresses gastric cancer cell proliferation and promotes apoptosis;  3. Fecal and serum SCFA (especially butyrate) levels are significantly low in GC patients. | (26) |
| SCFAs | CD8⁺ T cells | 1. Activate CD8⁺ T cells to produce IFN-γ and granzyme and further up-regulates tumor MHC I expression to reinforce immune responses; 2. Activate the cGAS/STING pathway by inhibiting histone deacetylases (HDACs) to induce DNA damage in colorectal cancer cells, up-regulating chemokines (CCL5, CXCL10) and ISGs | Colorectal cancer (CRC) | 1. In vitro studies demonstrate that SCFAs enhance tumor immunogenicity;  2. In vivo studies link higher SCFA levels with abundance of SCFA-producing gut microbiota. | (27) |
| SCFAs | CD8⁺ T cells | 1. Promote CD8⁺ T cell production of IFN-γ and granzyme B in an ID2-dependent manner, enhancing cytotoxicity and antitumor activity;  2. Up-regulate IL-12 receptor expression, boosting CD8⁺ T cell responsiveness to IL-12 and promoting effector function;  3. Enhance oxaliplatin chemotherapy efficacy | CRC, lymphoma, colitis-associated CRC | 1. Oral or intraperitoneal butyrate augments oxaliplatin efficacy in animal models;  2. Clinical data show higher serum butyrate in oxaliplatin-responsive cancer patients;  3. Preclinical combination of butyrate with anti-programmed cell death ligand 1 (PD-L1) immunotherapy enhances antitumor effects. | (28) |
| SCFAs | CD8⁺ T cells | Promote CD8⁺ T cell memory formation, modulates cellular metabolism, and sustains memory cell survival. | Indirectly participate in the tumor process | 1. In vitro studies demonstrate that CD8⁺ T cells treated with butyrate show stronger expansion and IFN-γ production;  2. High-fiber diet increases circulating SCFAs and enhances recall responses of memory CD8⁺ T cells. | (29) |
| SCFAs | CD4⁺ T cells | Inhibit CD4⁺ T cell activation and pro-inflammatory cytokine (IFN-γ, IL-17) production in a dose-dependent manner via HDAC inhibition and GPR43 activation, affecting Th1, Th17, and Th22 | Indirectly participate in the tumor process | 1. High-fiber diet increases butyrate and alleviates gut inflammation in animal models;  2. Fecal/tissue butyrate levels reflect intestinal immune homeostasis;  3. Butyrate enemas or HDAC inhibitors are under preclinical/early clinical investigation as adjuvant therapy for immune-checkpoint blockade (ICB) in IBD. | (32, 33) |
| SCFAs | Tregs | 1. Promote peripheral Treg generation;  2. Stabilize FOXP3 expression via HDAC inhibition and increase histone acetylation at the FOXP3 locus, enhancing Treg function | Indirectly participate in the tumor process | Dietary SCFA or prebiotic supplementation proposed as a theoretical basis for Treg modulation in autoimmune diseases. | (34) |
| Propionate | Tregs | Specifically expand colonic lamina propria Tregs, down-regulate CD69 expression, and promote Treg trafficking via draining lymph nodes and blood to sites of atherosclerosis. | Indirectly participate in the tumor process | SCFA supplementation (e.g., propionate) or modified starches to increase intestinal SCFA levels proposed as a preventive strategy for Abdominal Aortic Aneurysm at-risk populations. | (35) |
| SCFAs | Th17/Tregs | 1. Gut dysbiosis reduces propionate, skewing Th17/Treg balance (Th17↑, Treg↓);  2. Propionate supplementation restores Th17/Treg equilibrium by promoting Treg and suppressing Th17 differentiation via GPR43 activation and HDAC6 inhibition. | Indirectly participate in the tumor process | Propionate supplementation or microbiota modulation proposed as a novel immunomodulatory approach for chronic prostatitis/chronic pelvic pain syndrome (CP/CPPS). | (36) |
| SCFAs | Γδ T cells | Directly inhibit γδ T cell IL-17 and IL-22 production via HDAC inhibition | CRC | SCFAs (especially propionate) proposed as potential targets for modulating γδ T cell function in IBD and CRC. | (37) |
| SCFAs | B cells | Drive B cell differentiation into IL-10⁺ IgM⁺ regulatory plasma cells | Indirectly participate in the tumor process | Butyrate analogues under investigation as adjunct therapy for metabolic syndrome. | (45) |
| SCFAs | B cells | 1. Butyrate/propionate promotes the differentiation of IL-10⁺ IgM⁺ regulatory plasma cells and reduces pathogenic class switching via HDAC inhibition. 2. Acetate promotes the generation of Bregs and inhibits pro-inflammatory cytokines (e.g., TNFα); Butyrate reduces mitochondrial reactive oxygen species (ROS) in B cells via HDAC3 inhibition to maintain Breg homeostasis. 3. Butyrate induces the production of TGF-β and retinoic acid (RA), promoting IgA class switching in B cells and enhancing the intestinal barrier function. | CRC | 1. In vitro studies clarify the regulatory effects of SCFAs on B cell differentiation and antibody production. 2. HDAC inhibitors (e.g., butyrate analogs) reduce autoreactive plasma cells in animal models. | (46) |
| SCFAs | B cells | 1. Enhance B cell metabolism and provide energy and material basis for plasma cell differentiation;  2. Promote the production of IgA and IgG, enhancing the immune response against pathogens; 3. Indirectly regulate T cells by increasing the number of Tfh cells, promoting germinal center reactions, and assisting B cell antibody production. | Indirectly participate in the tumor process | 1. Animal studies have confirmed that a high-fiber diet/SCFA supplementation can enhance antibody levels and SCFAs regulate B cell functions through metabolic regulation and HDAC inhibition; 2. Antibiotic treatment can eliminate the antibody-promoting effect of SCFAs, confirming microbiota dependence. | (47) |
| Butyrate | Bregs | Promote IL-10 expression in Bregs, enhance their suppressive function, and inhibit germinal-center B cells and plasmablast differentiation | Indirectly participate in the tumor process | Butyrate supplementation alleviates intestinal inflammation. | (48) |
| SCFAs | Bregs | Enhance the suppressive function of Bregs, increase IL-10 secretion, and reduce the differentiation of plasmablasts, decrease the production of pro-inflammatory cytokines (TNFα, IL-6, MCP-1) | No associated cancers mentioned | 1. Verified the anti - inflammatory effect of butyrate supplements in animal models; 2. Fecal butyrate levels are decreased in rheumatoid arthritis patients and are positively correlated with peripheral blood Bregs. | (49) |
| SCFAs | B cells | Dose-dependently modulates B cells: low concentrations (50–200 µM) mildly increase AID expression and class-switch recombination (CSR); high concentrations (≥400 µM) inhibit AID, Blimp1, CSR, somatic hypermutation, and plasma cell differentiation | Indirectly participate in the tumor process | Modulating butyrate levels inhibits autoantibody production and alleviates lupus symptoms in animal models. | (50) |
| SCFAs | M2 Macrophages | 1. Trigger TLR3-induced autophagy in cancer cells, activating NF-κB and MAPK pathways and enhancing migration and invasion, autophagy induces CCL20 release;  2. CCL20 can recruit macrophages into the tumor microenvironment (TME) and polarizes them toward pro-tumor M2 Macrophages, further enhancing prostate cancer invasiveness. | Prostate cancer | 1. Preclinical studies establish SCFAs from Castration-Resistant Prostate Cancer-associated microbiota as key mediators linking dysbiosis to tumor progression;  2. CCL20 identified as a potential prognostic biomarker for prostate cancer. | (53) |
| SCFAs | Macrophages | 1. Inhibit LPS-induced M1 polarization (↓iNOS, TNF-α) and promote IL-4–induced M2 polarization (↑Arg-1, IL-10);  2. Down-regulate TLR4, MyD88, NF-κB, and suppress alcohol-induced liver injury | Indirectly participate in the tumor process | 1. Preclinical evidence shows inulin increases intestinal SCFAs; 2. SCFAs exert anti-Alcoholic liver disease (ALD) effects by modulating M1/M2 macrophage balance, providing rationale for inulin/SCFA-based ALD prevention and therapy. | (54) |
| SCFAs | Macrophages | 1. Modulate M1/M2 balance, ↓M1, ↑M2; reduce serum pro-inflammatory cytokines (IL-12p70, TNF-α, CXCL1);  2. ↑tight-junction proteins (ZO-1, occludin), restore barrier function | Indirectly participate in the tumor process | Positive correlations between SCFAs and bone-metabolism indices suggest novel gut-targeted osteoporosis therapy. | (55) |
| Acetate | Macrophages | 1. Promote pro-inflammatory M1 polarization (↑CD86, iNOS; ↓CD163, ARG1) via histone acetylation-driven ACC1 transcription and increase fatty-acid synthesis; 2. M1 macrophages enhance CD8⁺ T cell function (↑IFN-γ, granzyme B), increasing cytotoxicity against hepatocellular carcinoma (HCC) cells. | HCC | 1. Preclinical studies show *B.thetaiotaomicron*-derived acetate inhibits HCC growth via immune-microenvironment modulation;  2. Acetylation inhibitors (e.g., curcumin) block acetate-mediated tumor suppression, offering epigenetic-targeted HCC therapy. | (56) |
| SCFAs | Dendritic cells (DCs) | ↓ pro-inflammatory cytokine secretion (IL-6, IL-12) by DCs and  ↓ chemokines (CXCL9, CXCL10, CXCL11) | Potentially applicable to inflammation-associated cancers | Butyrate proposed as an anti-inflammatory agent for modulating DC function. | (61) |
| SCFAs | DCs | 1. Induce DC dendrite elongation via HDAC inhibition, promoting actin polymerization;  2. Enhance antigen uptake and presentation | Indirectly participate in the tumor process | Clinical application not yet addressed. | (62) |
| Butyrate | Myeloid-derived suppressor cells (MDSCs) | 1. Promote MDSC suppressive function via fatty-acid β-oxidation (FAO) metabolic reprogramming;  2. Enhances T cell inhibition by MDSCs | Indirectly participate in the tumor process | 1. Butyrate alleviates cholangitis in animal models;  2. Positive correlation observed between butyrate levels and MDSC function/treatment response in humans. | (68) |
| SCFAs | Natural killer (NK) cells | Promote the release of extracellular vehicles (EVs), significantly reduce the secretion of the anti - inflammatory cytokine IL-10, and indirectly weaken the pro - tumor effect of IL-10. | Multiple myeloma | 1. Enhancing the cytotoxicity of NK cells through SCFA preconditioning can optimize the effect of NK cell immunotherapy; 2. In combination with ICB, chemotherapy, etc., SCFAs may improve treatment response and reduce drug resistance. | (71) |
| SCFAs | Neutrophils | 1. Butyrate ↓CD66b, ↑CD16 and CD62L, yielding a low-activation, long-lived mature phenotype; propionate ↑CD54 and CXCR4, inducing a senescent phenotype;  2. Acetate and butyrate suppress neutrophil migration in vitro; propionate alters migratory phenotype (↑CD62L, CD54) without affecting migration. | Indirectly participate in the tumor process | In vitro studies demonstrate that pathological concentrations of SCFAs impair the anti-HIV function of neutrophils. | (72) |
| Butyrate, propionate | Basophils | 1. Induce CD69 expression and shift cytokine secretion (↓IL-4, ↑IL-13) via HDAC inhibition;  2. Induce basophil apoptosis even in the presence of IL-3 (apoptosis inhibition);  4. Enhance IgE-mediated degranulation | Indirectly participate in the tumor process | Mechanisms of HDAC-mediated basophil modulation by propionate and butyrate are clarified. | (73) |
| 3-oxoLCA, isoalloLCA | Th17/Tregs | 1. 3-oxoLCA directly bind the Th17 transcription factor RORγt, inhibiting its activity and reducing IL-17 secretion;  2. IsoalloLCA promotes mitochondrial ROS generation to induce Treg differentiation. | Indirectly participate in the tumor process | 1. Oral 3-oxoLCA reduces intestinal Th17 cells in animal models; 2. Combined 3-oxoLCA and isoalloLCA feeding increases Tregs and alleviates colitis in animal models. | (38) |
| Bile acids | CD8⁺ T cells | Inhibit CD8⁺ T cell function by enhancing PMCA activity, suppressing Ca²⁺-NFAT2 signaling, and reducing IFN-γ, TNF-α, and granzyme B secretion. | CRC | 1. Bile acid sequestrants (e.g., cholestyramine) lower DCA and inhibit tumor growth;  2. Fecal DCA concentration and microbial baiF gene (key for DCA synthesis) abundance are potential CRC risk biomarkers;  3. Polyamine blockade therapy combined with PD-1 inhibitors may reverse "cold tumor" microenvironment. | (39) |
| Secondary bile acids | DCs | Inhibit NF-κB activation via the TGR5–cAMP–PKA pathway, reducing secretion of pro-inflammatory factors (IL-1β, IL-6, TNF-α). | Indirectly participate in the tumor process | Oral DCA/LCA alleviates experimental autoimmune uveitis (EAU) in animal models. | (63) |
| Bile acids | MDSCs | 1. Promote MDSC infiltration into liver metastases and suppress T cell activation; 2. MDSC-derived CCL2 attenuates immunosuppression via CCR2 signaling. | Colorectal cancer liver metastasis (CRLM) | Potential targets (TGR5, CCL3, CCR1) proposed but remain preclinical. | (69) |
| Spermidine | CD4⁺ T cells | Inhibit CD4⁺ T cell via MAPK/ERK pathway, reduce activation marker CD69 and IL-2 production, decrease Th1 and Th17 differentiation | Indirectly participate in the tumor process | In vitro studies demonstrate that Spermidine show preventive and therapeutic effects, offering a potential strategy for multiple sclerosis requiring further preclinical and clinical validation. | (40) |
| L-Tryptophan (L-Trp) | Tregs | Promote Treg homing to the colon via the AhR-GPR15 pathway and increase colonic Tregs | Potentially reduce colitis-associated cancer risk | L-Trp supplementation is proposed as a non-invasive preventive therapy for ulcerative colitis (UC). | (42) |
| Indole-3-propionic acid (IPA) | Th1/Th17 | Bind HSP70, trigger mitochondrial-dependent apoptosis in Th1/Th17 cells | Potentially reduce IBD-associated CRC risk | Oral IPA alleviates colitis in animal models and is proposed as a therapeutic strategy for IBD. | (43) |
| Indole-3-acetic acid (IAA) | Macrophages | Induce IL-35 expression, promote Treg, Breg, and M2 macrophage differentiation, and inhibit Th1 differentiation. | Colitis-associated CRC | IAA levels are low in CRC patients, suggesting diagnostic or preventive value. | (59) |
| TMAO | M1 Macrophages | Activate NLRP3 inflammasome, promote mitochondrial ROS, activate NF-κB, induce M1 macrophage polarization, and enhance Th1 and Th17 differentiation | Relevant to Graft-versus-host disease (GVHD) after hematopoietic stem-cell transplantation for hematologic malignancies | Choline analogue can alleviate GVHD, suggesting therapeutic potential for dietary interventions or drugs targeting the TMAO pathway. | (57) |
| TMAO | Macrophages | Activate IFN-I pathway, promote M1 macrophage polarization, and enhance CD8⁺ T cell function | Pancreatic ductal adenocarcinoma, melanoma | 1. Higher TMAO levels correlate with improved long-term survival and immunotherapy response;  2. Dietary choline supplementation or adoptive transfer of TMAO-conditioned macrophages shows therapeutic potential. | (58) |
